# Beyond bikini medicine: An analysis of  Sex- and Gender-Informed Medicine in a preclinical undergraduate medical education.

**DOI:** 10.12688/mep.20959.1

**Published:** 2025-08-08

**Authors:** Katherine Wasden, Zoé Kibbelaar, Celeste S Royce, Natasha R Johnson, Alex S Keuroghlian, K. Meredith Atkins, Deborah Bartz

**Affiliations:** 1 Harvard Medical School, Boston, Massachusetts, 02115, USA; 2 Department of Obstetrics, Gynecology and Reproductive Sciences, Magee-Womens Hospital, University of Pittsburg School of Medicine, Pittsburgh, PA, USA; 3 Department of Obstetrics, Gynecology and Reproductive Biology, Beth Israel Deaconess Medical Center, Boston, Massachusetts, 02115, USA; 4 Department of Obstetrics, Gynecology and Reproductive Biology, Brigham and Women's Hospital, Boston, Massachusetts, 02115, USA; 5 Department of Psychiatry, Massachusetts General Hospital/Harvard Medical School, Boston, Massachusetts, 02115, USA

**Keywords:** Medical school, women’s health, sex, gender, LGBTQIA

## Abstract

**Introduction:**

Despite the expanding literature demonstrating widespread sex and gender differences across all organ systems, the inclusion of Sex- and Gender-Informed Medicine (SGIM) in medical education is lacking, leaving medical students without an appreciation for physiologic and sociocultural differences that affect health, disease, and healthcare delivery.

**Methods:**

We performed an audit of the five courses of the Harvard Medical School pre-clinical curriculum that teach physiology and pathophysiology using case-based collaborative learning (CBCL). Using a standard codebook, reviewers recorded: time per case, diagnosis/focus of case, age, sex, gender, pronouns, pregnancy status, and sexual orientation. Coders were asked to determine if the CBCL patient’s sex/gender chosen was “intentional” and if there was further discussion around sex- and gender-specific influences on disease. Each case was coded by two auditors, with discrepancies adjudicated by a third.

**Results:**

Across five courses, 591 patient cases taught over 380 hours were analyzed. 298 (50.4%) of CBCL patients were women, 235 (39.8%) men, five (0.8%) non-binary, and 53 (9.0%) gender undefined. Relationships were predominantly between discordant gendered partners (37 cases, 82.2% of cases with relationships). Only 60 cases (10.2%) were coded as having a patient sex or gender that was intentionally chosen during case design, predominantly to reflect population-level disease epidemiology or prevalence by gender (45%). Only 39 cases (6.6%) included deliberate learning dedicated to sex or gender differences in health and disease, with most discussions within cases of reproduction or inflammatory/autoimmune diseases.

**Conclusion:**

Our review demonstrated a deficiency of SGIM content in our institution’s preclinical curriculum with only 6.6% of CBCL cases providing deliberate teaching on sex and gender differences in health and disease, largely confined to reproductive topics. We propose the creation of dedicated, daily course material in collaboration with experts to increase exposure to SGIM so students can confidently treat any patient.

## Introduction

Motivated by a goal of empowerment and liberation, second-wave feminists in the 1960s and 1970s began the Women’s Health Movement as a means of better understanding their bodies and their health ^1^. However, the scientific and medical communities were not well poised to satisfy this intellectual curiosity, especially given that less than 6% of medical students were women during this time period ^2^. Women were excluded from clinical trials as a paternalistic protection against the possibility of study interventions causing fetal harm ^3^ and due to unfounded concerns that monthly hormonal fluctuations would muddle study results ^2– 4^. It was only in 1993 that the FDA withdrew its ban on the inclusion of reproductive-aged women in Phase 1 and 2 clinical trials through the NIH Revitalization Act, requiring inclusion of women and under-represented groups as research subjects in clinical trials ^1, 4^. While approximately half of the participants in NIH-funded studies were women as of 2015 ^5– 7^, the following year a study of published NIH-funded randomized controlled trials reported that less than a third of studies in the cohort had actually analyzed their data by sex as a means of better understanding sex-based differences in research outcomes ^5, 8^. Compared to the relative disease burden, NIH funding dedicated to diseases that predominantly affect women has always been and remains woefully underfunded today ^6, 7, 9^. This lack of research inclusion, scientific rigor, and appropriate funding contributes to the persistence of a male standard in medical literature, education, and clinical care ^10^. Even when well-designed research has led to advances in our understanding of sex differences in disease presentation, pathogenesis, and response to treatment, this knowledge often does not find its way into our medical classroom. As a result, physicians are rarely educated to tailor their practice to the uniqueness of the patient in front of them, leading to adverse health outcomes for women and sex- and gender-minorities ^10– 12^.

Sex- and Gender-Informed Medicine (SGIM) is a framework which integrates knowledge of the impact of sex and gender on health and disease into clinical practice, from health risk to disease treatment ^11^. In this framework, “sex” is understood as biological and physiological characteristics assigned at birth and typically categorized into male, female, and intersex; “gender” is understood as a spectrum of socially and culturally influenced and constructed roles, behaviors, and identities ^11^. Medical education rarely incorporates this sex- and gender-informed framework into curricula or clinical care. Some attribute this deficit to a lack of clear course learning objectives and assessment requirements, such as inadequate incentive due to a dearth of SGIM content inclusion on licensing exams ^13, 14^. A 2011 survey of faculty representing 44 medical schools in the United States and Canada, demonstrated that 70% of schools did not have formal curricular requirements for the inclusion of SGIM, and up to 70% of respondents reported minimal, if any, SGIM content ^13– 15^. A subsequent survey of over 1,000 U.S. medical students who participated in national medical student organizations found 96.0% believed taking sex and gender into account improves patient care and 94.4% believed SGIM should be in the curriculum. In contrast, only 31.1% reported having such a curriculum in their school and only 34.5% reported they would be prepared to incorporate sex and gender difference in their practice ^13^.

To consider curricular solutions, it is crucial to understand in a more granular, systemic manner where SGIM is currently included in the medical school curriculum and where opportunities exist for expanding this content. We, therefore, conducted an audit of our own preclinical curriculum (one academic year) with the primary objective of analyzing the manner and degree of SGIM topic inclusion for all first-year undergraduate medical students. The findings from this audit identify curricular gaps in SGIM content, allowing for specific and actionable suggestions for novel curricular design improvements to newly incorporate SGIM material in all relevant health topics.

## Methods

In this observational study, we performed a curricular audit of the first-year Harvard Medical School (HMS) Pathways preclinical academic year from August 2022 to August 2023 ^16^. We chose the preclinical curricular year to study because it is the only time when the entire class has the same required classroom content before starting clerkships at four different HMS-affiliated hospitals in year two and elective courses in years three and four. This first-year classroom curriculum is grounded in a cooperative learning pedagogy, called case-based collaborative learning (CBCL) ^17, 18^. The CBCL small-group discussions are situated on learning theory that posits interactive activities specifically around patient cases, designed by faculty and discussed with peer learners, driving knowledge acquisition through shared reflections, probing questions, and shared critical thinking ^19^. Our audit reviewed all patient cases used in the HMS preclinical CBCL curriculum.

Five of seven preclinical courses run over the year primarily relied on CBCL and were grouped for analysis as: 1) biochemistry, anatomy, genetics, immunology 2) dermatology, infectious disease, rheumatology 3) cardiology, pulmonology, hematology 4) gastroenterology, nephrology, endocrinology, and 5) neurology, and psychiatry (referred to moving forward as “Courses 1–5”). The two excluded courses had pedagogical goals not focused on teaching physiology and pathophysiology but instead teaching medical ethics, healthcare policy, and practical clinical skills to prepare students to transition from the classroom to the clinical learning environment. Additionally, any non-CBCL learning activities within the five included courses, such as patient clinics and anatomy lab presentations, were also excluded.

Eleven student reviewers audited all eligible materials using a codebook developed *a priori*, with agreed-upon definitions for data coding ( Table 1). All reviewers participated in training meetings where pilot, standardized cases were analyzed together to establish group norms and to modify the codebook iteratively. All reviewers received access to the entire academic year 2022–2023 preclinical curricular content on the HMS web-based learning management system. An eligible CBCL case required a patient clinical scenario with at least one aspect of patient identity provided as part of the case. Aspects of patient identity assessed include age, sex, gender, pronouns, sexual orientation in the setting of an intimate partnership, and pregnancy status ( Table 1). We assessed the class time allotted per case (determined by dividing the total class time by the number of cases done in that class) and recorded the final patient diagnosis.

**Table 1.** Codebook Definitions Utilized by Reviewers. Acronyms: Case-based collaborative learning (CBCL), sex- and gender-informed medicine (SGIM).

Data Term	Definition
Designated time	Time calculated per case (number of CBCL cases divided by total class time dedicated to CBCL)
Teaching diagnosis or focus of learning	What was the final diagnosis or focus of learning (if no official diagnosis is given) of the case?
Age	As stated specifically in the case, typically in years for children and adults and months in infants.
Sex	Stated explicitly as male, female, intersex, or sex assigned at birth. If unspecified, biological and physiological references such as anatomy, chromosomes, or multiple secondary sex characteristics, can be used.
Gender	Gender identity explicitly stated in a sentence or in introduction of the patient as man, woman, or non-binary (or can utilize pronouns as presumption of gender). Non-binary defined as explicit statement that patient uses they/them pronouns or explicit mention in the case that patient identifies as non-binary.
Pronouns	Pronouns used for the patient throughout the case text.
Pronouns specified	Yes: pronouns are explicitly stated either in an introductory sentence (eg. Patient uses ____ pronouns) or in parenthesis in the introduction of the patient; implication that pronouns used were provided by the patient (e.g. Patient Jane ( *she/her*) is a 30-year-old…).
Intercourse	Is sexual intimacy mentioned or implied as having impacted the patient’s health in the case?
Sexual Orientation	If a romantic or intimate partner is mentioned in the case, is the partner’s gender the same (concordant) or different (discordant) than the patient’s gender, or is it not mentioned (unspecified).
Sex/Gender “intentional”	By coder impression, was the sex/gender of the patient integral to the content of the case or believed to be specifically chosen by the faculty in case development (Yes)? Or was patient sex/ gender irrelevant to the medical content of the case (No)?
“Deliberate” SGIM teaching	Did the case include an explicit mention, even minor, of the impact of sex and/or gender on health related to the medical condition discussed in the case?
SGIM Teaching Content Area: - Disease Presentation - Statistics - Anatomy - Physiology - Response to Treatment - Risk Factor - Socioeconomic Factors - Interaction with the Healthcare System	If “yes” to deliberate teaching above: Is there a discussion of differences in disease presentation between sex and gender? Are there statistics about the disease broken down by sex or gender? Are anatomical differences by sex discussed? Are physiological differences by sex discussed? Are differences in response to treatment discussed? Is sex or gender discussed as a risk factor? Are socioeconomic differences between sexes/genders discussed as these relates to their health and care? Is there a discussion of the patient's engagement or interaction with the healthcare system as it impacts their health and care?

Next, we assessed if the CBCL patient’s sex/gender designation was perceived as “intentionally” assigned by the faculty case creator due to some known or taught information regarding sex or gender differences in disease risk, prevalence, presentation, or course. For example, 45.0% of the 60 cases we coded as intentional were given that designation because the patient’s gender correlated with a higher prevalence of the disease by gender in the U.S. population; approximately 80% of autoimmune diseases are diagnosed in U.S. women ^20^, and 63% of autoimmune CBCL cases were presented in women patients. Similarly, 75% of U.S. patients diagnosed with autism spectrum disorder are boys ^21^ and 100% of the CBCL cases on autism were presented in CBCL cases with boys. While this may reflect anchoring bias on the part of the reviewer, we wanted to flag these cases as opportunities for SGIM teaching content in the case discussion as well as for curricular growth to combat this bias.

Lastly, we assessed whether further SGIM discussion was pursued in class materials. These cases were coded as having “deliberate” teaching dedicated to SGIM with specific content on sex or gender differences in health and disease. Those cases with deliberate teaching were further categorized by the content areas that was covered (e.g., the impact of sex and/or gender on disease risk versus treatment versus interactions with the healthcare system, with a complete list of content areas in Table 1). For example, a case that includes a report that patients who are men have a worse prognosis with schizophrenia compared to women would be listed as a case with deliberate teaching on gender as a “Risk Factor.”

Each CBCL case in the audit was analyzed by two reviewers, and a third reviewer adjudicated discrepancies. Given the potential for greater subjectivity, all cases marked as having intentional patient sex/gender or providing deliberate sex/gender teaching were automatically reviewed by a third reviewer who re-analyzed the original case and recorded the content areas ( Table 1) that they interpreted as presented in the case. Descriptive statistics were performed using Excel (Version 16.51, 2021 Microsoft).

## Results

### Overview of course materials

Across the five eligible courses, 591 distinct CBCL patient cases taught over 380 hours were eligible and analyzed ( Table 2): 123 (20.8%) in Course 1, 63 (10.7%) in Course 2, 142 (24.0%) in Course 3, 141 (23.9%) in Course 4, and 122 (20.6%) in Course 5. Across the 591 patient cases, 470 unique diseases were presented as learning topics. Patient ages spanned across the life course: newborn (zero days – one month; 7 cases, 1.2%), infant (one month – one year; 15 cases, 2.5%), child (one year – 12 years; 40 cases, 6.8%), teenager (13 years – 17 years; 31 cases, 5.2%), adult (18 years – 64 years; 321 cases, 54.3%), and elderly (> 65 years; 114 cases, 19.3%), with the remaining (63 cases, 10.6%) without a listed age in the case.

**Table 2.** Overall Case Distribution Across Curriculum. Case distribution by course, patient age, and patient gender represented as absolute number of cases, and then percentage of the total cases (N=591).

	All Cases (N=591)
**Course**	
Course 1	123 (20.8%)
Course 2	63 (10.7%)
Course 3	142 (24.0%)
Course 4	141 (23.9%)
Course 5	122 (20.6%)
**Age**	
Newborn (zero days – one month)	7 (1.2%)
Infant (one month – one year)	15 (2.5%)
Child (one year – 12 years)	40 (6.8%)
Teenager (13 years – 17 years)	31 (5.2%)
Adult (18 years – 64 years)	321 (54.3%)
Elderly (> 65 years)	114 (19.3%)
Unknown	63 (10.6%)
**Gender**	
Woman	298 (50.4%)
Man	235 (39.8%)
Non-binary	5 (0.8%)
Unknown	53 (9.0%)

### Patient gender, sex, and sexuality

Gender, rather than sex, was almost ubiquitously used to identify patients in cases. The distribution of patient gender included 298 CBCL cases (50.4%) where the patient identified as a woman, 235 (39.8%) as a man, five (0.8%) as non-binary, and 53 (9.0%) where the gender was undefined. This distribution was relatively consistent across all five courses. Using the codebook definition ( Table 1), patient sex was not identified in most cases (580, 98.1%), with a total of four cases (0.7%) of male- and seven cases (1.2%) of female-identified patients. No transgender patients were represented in the curriculum. There was one patient case of a newborn with variations in physical sex development, though there was no explicit use of the term “intersex,” and no sex or gender identifiers were given. Only 16 CBCL cases (2.7%) specified upfront that a patient used particular pronouns. Intimate partners were mentioned in 45 cases (7.6%). The majority of these were between partners of discordant gender (37 cases, 82.2%). Four cases (9%) included partners of concordant gender and four (9%) were unspecified.

Only 16 cases (2.7%) in the preclinical curriculum featured pregnant or recently pregnant patients. Of these, only five cases, less than 1% of the entire curriculum, focused on diseases specific to pregnancy (normal physiology of pregnancy, mastitis, gestational hypertension, pre-eclampsia, preterm premature rupture of membranes); the remaining 11 cases were explorations of the impact of pregnancy on other diseases such as autoimmune thyroiditis, anemia, or thromboembolic risk. In addition, two cases focused on the use of contraception and two focused on infertility. All patients in these pregnancy cases were cisgender women with either unknown or gender-discordant partners. Overall, 13.95 hours of 380 hours (3.7%) of the entire preclinical curriculum were dedicated to pregnancy and reproduction.

**Figure 1.**
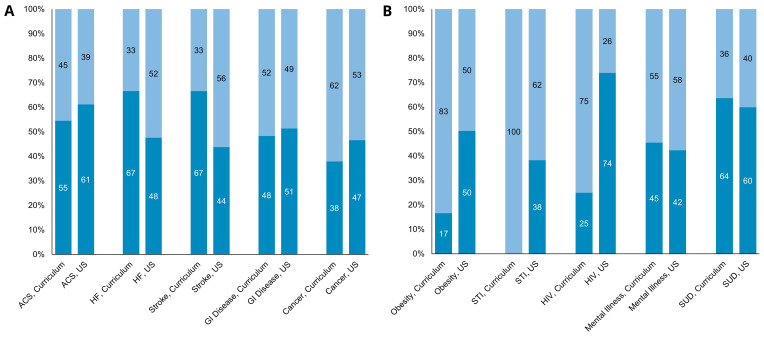
Disease Distribution by Gender of Health Conditions in School Curriculum Compared to U.S. Disease Prevalence. X-axis lists the disease categories, and the Y-axis reflects the prevalence of the disease by percentage. Numbers listed in columns reflect prevalence by gender (percentage). Women patients in light blue, men patients in dark blue. Relative prevalence of disease in the HMS preclinical curriculum is the left column, followed by the relative prevalence of the disease in the United States. ( **A**) Gender Distribution of Non-Stigmatized Diseases. Acronyms: Acute coronary syndrome (ACS), heart failure (HF), gastrointestinal (GI). ( **B**) Gender Distribution of Stigmatized Diseases. Acronyms: substance use disorder (SUD), sexually transmitted infection (STI).

Diseases that had a high frequency of repetition in cases, such as heart failure, stroke, gastrointestinal disease, and cancer, reflected a gender distribution within the pre-clinical curriculum that was similar to the distribution of the disease prevalence by gender within the United States ( Figure 1A) ^22^. However, diseases that carry cultural stigmatization, specifically obesity, STIs excluding HIV (100% of these cases were in female patients) and HIV, showed a strong woman bias within the curriculum as compared to the U.S. national population prevalence ( Figure 1B) ^22, 23^.

### Intentional designation of patient sex and gender in curricular cases

**Figure 2.**
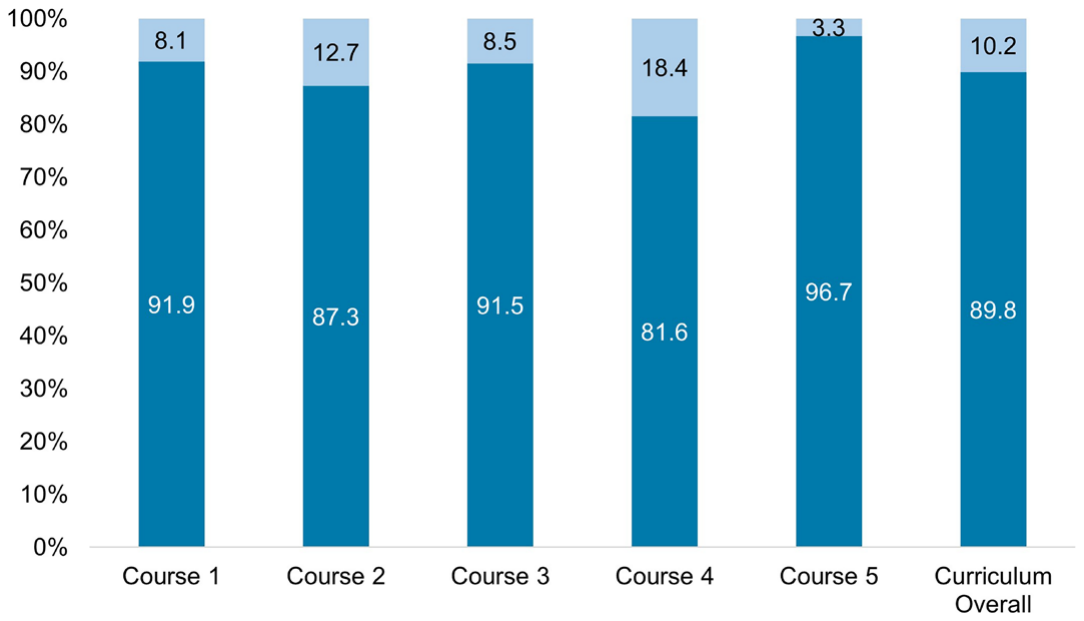
Distribution of Intentionally Assigned Sex and Gender within Cases Across the Pre-Clinical Curriculum. Dark blue represents the percent of cases where the patient sex or gender was perceived as intentionally assigned. Light blue represents the percent of cases where the patient sex or gender perceived as intentionally assigned. The total number of cases in curriculum overall (far right) was 591, with 60 (10.2%) coded as intentionally having patient sex and gender assigned, represented in light blue. Courses are 1) biochemistry, anatomy, genetics, immunology 2) dermatology, infectious disease, rheumatology 3) cardiology, pulmonology, hematology 4) gastroenterology, nephrology, endocrinology, and 5) neurology and psychology.

We coded 60 of 591 cases (10.2%) as having a patient sex or gender that was intentionally chosen by faculty during case design, distributed evenly throughout the five courses ( Figure 2). In these cases, 45 of the patients were women (75.0%), 13 were men (21.6%), one was non-binary (1.7%), and one was undefined (1.7%; this was a case of congenital adrenal hyperplasia where the patient had variations in physical sex development). Twenty-seven cases (45.0%) were perceived as intentional because the patient gender correlated with a higher prevalence of the disease in that gender in the U.S. population. The teaching associated with 16 of these 27 cases (59.3%) provided no mention of the increased prevalence by sex/gender or any further information on physiologic sex or sociocultural differences that would explain why that differential exists, how it manifests in gender differences in disease presentation, or what the significance might be for disease progression or treatment. Twenty of the 60 cases (33.3%) were coded as intentionally assigned a sex/gender to correspond with teaching content dedicated to sex-specific anatomical or physiological aspects of a disease, for example, a female patient with the absence of menses as a symptom used to help diagnose Graves’ disease. Eighteen cases (30.0%) with teaching on pregnancy, reproduction, and contraception were coded as intentionally being situated with women patients. Finally, three cases (5.0%) were coded as intentional due to further teaching related to gender differences in healthcare delivery, such as the historical restrictions on blood donation for men having sex with men.

### Deliberate discussion of sex and gender in curricular cases

**Figure 3.**
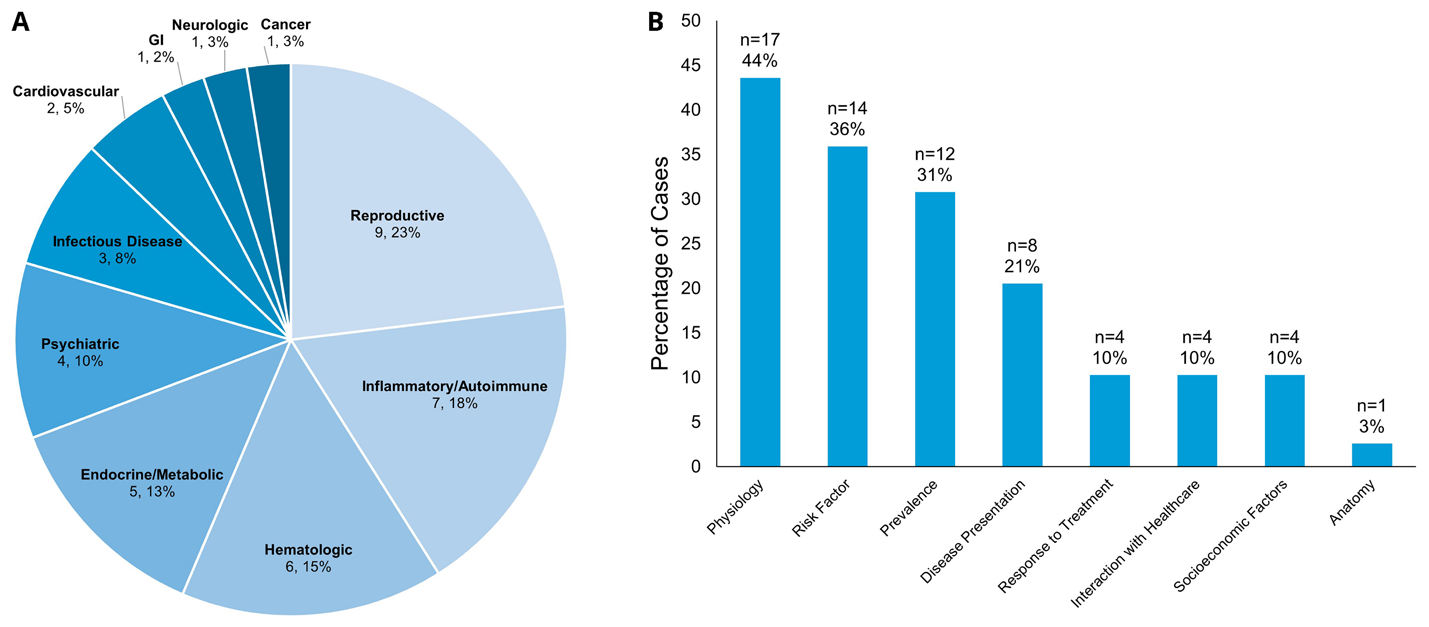
( **A**) Breakdown of Cases with Deliberate SGIM Discussion by Medical Topics. Numbers reflect the number of cases within each medical topic and percent of the total of 39 cases with deliberate SGIM discussion. ( **B**) Deliberate Discussion Content Areas ( Table 1). The X-axis reflects the content area within the CBCL cases that had a SGIM discussion. The Y-axis reflects the percentage of cases with deliberate discussion within each content area of the total 39 cases with deliberate SGIM teaching (categories not mutually exclusive).

Ultimately, only 38 of the 60 cases (63.3%) the coders perceived as having an intentional assignment of sex/gender had any deliberate further teaching on sex and gender differences in the specific health condition. One more case that had not been coded as having an intentional sex/gender assignment was coded as incorporating deliberate teaching regarding gender impact on care that was not directly related to the patient in the CBCL case. Therefore, a total of 39 of the 591 cases (6.6%) had formal SGIM teaching. In these cases, 28 of the patients were women (71.8%), nine were men (23.1%), and two were non-binary (5.1%). These SGIM discussions occurred most frequently when the diagnosis was related to reproduction (9 cases, 23.1%), inflammatory or autoimmune disease (7 cases, 17.9%), hematologic disease (6 cases, 15.4%) or an endocrine or metabolic disease (5 cases, 12.8%) ( Figure 3A). When looking at the content areas ( Table 1) discussed in cases with teaching on sex- and gender differences, the most common areas of SGIM deliberate content included physiology (17 cases, 43.6%), risk factors for disease (14 cases, 35.9%), and prevalence of the disease (12 cases, 30.8%) ( Figure 3B). Of the 470 unique diseases covered in the curriculum, 435 of them (92.6%) did not integrate SGIM into the class content.

## Discussion

Despite the expanding literature demonstrating widespread sex and gender differences across all organ systems ^11^, our review demonstrated only 6.6% of the CBCL cases that anchor our institution’s preclinical learning make any mention to sex and gender differences in biochemistry, anatomy, genetics, physiology, pathophysiology, or psychology. A similar 2016 audit performed at Texas Tech University Health Sciences Center identified that, compared to an ideal SGIM curriculum informed by Legato’s *Principles of Gender-Specific Medicine*, their curriculum only covered 41% of the recommended topics in the first-year and 60% of the recommended topics the second-year of school ^24^. A student led audit at Yale Medical School in 2019 demonstrated that less than 25% of their didactics discussed the impact of sex or gender on disease or medical outcomes, and only 8.1% of sessions had an in-depth discussion of SGIM, predominantly focused on physiology or disease prevalence ^25^.

When cases specific to pregnancy and reproduction, often referred to as “bikini medicine,” are removed from the cohort the frequency of SGIM content drops to just 5.1%. Already, only 7.4% of the 470 diseases covered in the pre-clinical curriculum contain SGIM content. Diminishing the discussion of SGIM to medical conditions associated with reproduction perpetuate the antiquated belief that physiologically, women do not significantly differ from men beyond reproduction. However, we are not arguing that there is too much reproduction or pregnancy teaching in our preclinical curriculum; pregnancy and reproduction related topics made up only 3.4% of the cases of the curriculum, despite being the focus of up to 12% of STEP 2 CK exam questions ^26^ and despite the six-week OBGYN clerkship making up 12.5% of the principal clerkship year. Thus, women are doubly disadvantaged by our curriculum, with little total body SGIM and a disproportionately small amount of content related to the women-specific pregnancy and reproduction topics critical to the health of close to six million U.S. patients and families each year. This is particularly alarming (and potentially causally related) given that, despite having the highest spending on healthcare, U.S. maternal mortality rates remain the highest of high-income countries ^27^.

While the minimal amount of SGIM teaching alone was disappointing, findings within our audit provided an aggregated view of our curriculum that calls for specific curricular change; factors such as the over-representation of women in stigmatized diseases, the lack of attention to anatomical or physiological differences in content topics, and the paucity of discussion of social, cultural or economic considerations suggests room for substantial improvement in the quality of curricular content. While we did not formally assess the complexity or depth of content related to SGIM in this analysis, we found only a single case that included a layered, comprehensive discussion of SGIM, dedicated to the historical underrepresentation of women in cardiac trials and the detrimental effect this exclusion has had on treatment and diagnosis of acute coronary syndromes in women.

With the growing recognition of the need for formal SGIM curricula in medical education, the 2020 Sex and Gender Health Education Summit drafted four tenets to guide education of medical professionals in SGIM: 1) Demonstrate knowledge of sex and gender, 2) Evaluate literature and the conduct of research for incorporation of sex and gender, 3) Incorporate sex and gender considerations into decision making, and 4) Demonstrate patient advocacy with respect to sex and gender ^14^. These guidelines are designed to serve as a foundation for educational efforts, ensuring that students and professionals have an appreciation for and ability to integrate SGIM into their practice.

Even with growth in our understanding of SGIM, integration into medical education is limited by varying levels of understanding and awareness of SGIM as a body of literature and as a discipline, both by individual course directors and institutional leadership. We must continue to advocate for and disseminate our expanding scientific understanding of SGIM, ensuring that rigorous research standards support exploring new knowledge. Faculty may believe that they do not have the expertise to teach sex- and gender-specific content in medical education if they themselves have not received education on the topic ^28^, continuing generational cycles of gender-biased medical education. Additionally, faculty often cite time restrictions as a barrier to creating and including new material. The multitude of barriers can be a daunting challenge to curricular improvement.

Our own initial effort to improve SGIM in the overall medical school curricula included developing a month-long selective course for junior and senior students within the roster of HMS advanced integrated science courses ^29, 30^ called *Sex- and Gender-Informed Medicine* ^31^. While popular, with a waitlist of students eager to join the course every year since its start in 2021, it is ultimately capped by institutional requirements at just 25 students who can learn this content a year. These students provide frequent feedback reflecting dismay at not having learned this SGIM material earlier in the curriculum. Our next response will be to engage SGIM experts at our HMS-affiliated hospitals to collaboratively craft one to four content slides for each CBCL case or syllabus, deliberately weaving focused, relevant, daily SGIM longitudinal content related to the disease or health condition taught throughout the preclinical curricular phase. We are aware of one other medical school that has similarly created a freely available slide library dedicated to SGIM content in a wide variety of health topics, freely available in a slide library ^32^. This longitudinal, interwoven approach may be more effective than a SGIM-specific class or course ^33^.

Our study is limited as we tried to retrospectively assess the intentionality of gender assignment within CBCL patient cases. While we erred on being inclusive when assigning intentionality to capture the greatest potential volume of SGIM within the curriculum, we may have misperceived the intentionality of case writers within our review. We aimed to minimize this potential for information bias through our rigorous codebook development and refinement, coder training with pilot cases, and utilization of at least two coders for most outcomes and a third reviewer for assessment of intentionality and deliberateness. Our inclusion of the five courses that used CBCL did exclude analysis of two courses, both of which have greater focus on social medicine, where discussions around the health impact of patient sex and gender may have been more prevalent. However, it is crucial that discussions of SGIM are not only taught through a “social medicine” lens but are instead integrated throughout pathophysiology teaching in medical school curricula. Finally, we did not audit discussions of SGIM which may have occurred in the classroom but were not documented in the online materials we screened.

In an era that recognizes precision medicine as a means of more effectively tailoring medicine to the individual patient, the lack of consideration of the role of sex and gender in physiology and disease has led to disparate health outcomes and, indeed, direct harm to patients. For example, women have a 1.5–1.7 fold greater risk of adverse drug reactions, while the different initial presentations of cardiovascular diseases compared to men (stroke, heart failure, coronary artery disease), results in longer door-to-door imaging times for diagnosis of stroke ^11^. When these differences are not widely taught in medical school, early career physicians are left without an appreciation for the physiological differences of which they should be aware and may be more likely to make diagnostic errors from cognitive bias. Our preclinical, case-based curriculum review identified the scarcity of detailed and thoughtful SGIM content taught within our institution’s pre-clerkship curriculum. We propose steps for future development of this material to prepare students to appropriately diagnose and treat every patient, utilizing medical knowledge that reflects each patient’s broader uniqueness.

## Ethics and consent

Ethical approval and consent were not required as this study was not human research.

## Data Availability

Dryad: Audit of SGIM in medical school pre-clinical curriculum https://doi.org/10.5061/dryad.djh9w0wbr ^34^ The project contains the following underlying data: variables collected from all cases as collected in the codebook. This work is licensed under a CC0 1.0 & Creative Commons Attribution 4.0 International (CC BY 4.0) License.
