# Supplementary material for: Beyond bikini medicine: An analysis of  Sex- and Gender-Informed Medicine in a preclinical undergraduate medical education
Source: MedEdPublish (2016). 2026 Jan 9;15:45. Originally published 2025 Aug 8. [Version 2] doi: 10.12688/mep.20959.2 (PMC12891956; doi:10.12688/mep.20959.2)
Supplement: Peer review history

# Reviewer report: Reviewer response for version 2

**DOI:** 10.21956/mep.22960.r45192

**Published:** 2026-02-12

**Authors:** Sowbhagya Micheal

**Affiliations:** 1 Western Sydney University, South Penrith, New South Wales, Australia

This well-written and coherent article highlights both the significance of teaching Sex and Gender Informed Medicine (SGIM) in medical school and also the current inadequacy of its integration within medical school curricula. The authors describe an audit of first year pre-clinical curriculum that focuses on physiology and pathophysiology delivered using case-based collaborative learning. The primary objective of the audit was to analyze the ways and extent to which SGIM in incorporated in pre-clinical curriculum. The audit methods and codebook are clearly explained and sufficiently detailed to allow replication in other medical school settings. Authors appropriately note the potential for anchoring bias on the part of the reviewers. Results indicate limited integration of SGIM in the pre-clinical medical curriculum and identify critical curricular gaps in SGIM content. These include over-representation of women in stigmatized diseases, lack of attention to physiological or anatomical differences and lack of adequate discussions on social, cultural and economic considerations. Results also illustrate opportunities to develop specific, actionable curricular improvements such as engaging SGIM experts to collaboratively develop SGIM content slides for each case-based collaborative learning case scenario.

 Suggestions to strengthen the article:

1. The paper discusses the audit being conducted by eleven student reviewers audited all eligible materials using a codebook developed a priori. It would be helpful to indicate which stage of medical training these students were undertaking during the audit process. In particular, please specify if any of these student reviewers were in their first year of the undergraduate program.
2. While the overrepresentation of women in stigmatized diseases and its implications is noted in the Discussion section, other critical findings of the audit, namely lack of teaching on transgender health, intersex conditions and minimal mention of non-binary identities are not mentioned. Please add some thoughts on how you intend to address these gaps in your pre-clinical curriculum.
3. The authors note important challenges in SGIM curricular improvements such as lack of expertise in faculty members and time constraints noted. Please indicate in your Discussion section whether you have developed strategies to overcome these barriers to improve SGIM teaching.

Have any limitations of the research been acknowledged?

Yes

Is the work clearly and accurately presented and does it cite the current literature?

Yes

If applicable, is the statistical analysis and its interpretation appropriate?

Yes

Are all the source data underlying the results available to ensure full reproducibility?

No

Is the study design appropriate and is the work technically sound?

Yes

Are the conclusions drawn adequately supported by the results?

Yes

Are sufficient details of methods and analysis provided to allow replication by others?

Yes

Reviewer Expertise:

Community-engaged medical education, social determinants of health, gender and sexuality, inclusive curriculum

I confirm that I have read this submission and believe that I have an appropriate level of expertise to confirm that it is of an acceptable scientific standard.

# Reviewer report: Reviewer response for version 2

**DOI:** 10.21956/mep.22960.r45232

**Published:** 2026-02-10

**Authors:** Kat JD Butler

**Affiliations:** 1 University of Toronto, Toronto, Canada

Review

 Summary:

 This is a clearly written paper outlining an audit of current case-based collaborative learning materials in the first year preclinical courses at Harvard Medical School, with an analysis focused on sex- and gender-informed medicine. The authors found little intentionality in curriculum design regarding SGIM, and describe some interventions they propose locally to address this lack of intentionality. 

 Methods

 The methods are clearly described and appropriately rigorous for the goals described, and in particular the use of a third reviewer to examine intentionality and deliberateness of teaching. 

 I answered 'partly' regarding whether all materials were freely accessible as I am not certain from the paper that the curricular content they analyzed is freely accessible.

 Results

 The results are clearly described and appropriately contrasted to national-level data regarding prevalence by gender. The histograms illustrate their points well. 

 Discussion

 The authors draw appropriate links to the significant issues related to under-teaching of SGIM at the undergraduate level in the USA, in particular highlighting care disparities for women and a significant rate of maternal mortality for a high income country. 

 I would suggest that one limitation of this study inherent to its focus on sex and gender is the fact that several of these disparities in outcome are shaped by intersectionality - Black women face higher rates of maternal mortality, for example. While it is essential to examine the inclusion and intentionality around SGIM teaching, the discussion may be a place to acknowledge that looking at the effects of SGIM without also examining under-teaching or pathologized teaching about racialization, financial stability, access to care, etc, are of course also factors that intersect with sex and gender to shape care outcomes. This study is valuable in and of itself, and the examination of how and whether physiological differences related to sex and gender are taught is crucial, and it is would also be beneficial to highlight the role of intersectionality here.

 I am adding a citation below which may be helpful in your discussion of the importance of including teaching about sex and gender throughout the curriculum. 

 Thank you for your work on this informative and valuable paper!

Have any limitations of the research been acknowledged?

Yes

Is the work clearly and accurately presented and does it cite the current literature?

Yes

If applicable, is the statistical analysis and its interpretation appropriate?

Not applicable

Are all the source data underlying the results available to ensure full reproducibility?

Partly

Is the study design appropriate and is the work technically sound?

Yes

Are the conclusions drawn adequately supported by the results?

Yes

Are sufficient details of methods and analysis provided to allow replication by others?

Yes

Reviewer Expertise:

Trans health, qualitative methods, anesthesia

I confirm that I have read this submission and believe that I have an appropriate level of expertise to confirm that it is of an acceptable scientific standard.

## References

[1] : Integrating trans health knowledge through instructional design: preparing learners for a continent – not an island – of primary care with trans people. *Education for Primary Care* .2021;32(4) : 10.1080/14739879.2021.1882885 198-201 10.1080/14739879.2021.1882885 33568022

# Reviewer report: Reviewer response for version 1

**DOI:** 10.21956/mep.22448.r43967

**Published:** 2025-11-08

**Authors:** Maricedes Acosta Martinez

**Affiliations:** 1 Renaissance School of Medicine at Stony Brook, Stony Brook, UK

This article describes the findings from an audit performed on the curriculum for first year medical students to determine the extend at which Sex- and Gender Informed Medicine (SGIM) was integrated in the physiology classes (Courses 1-5). The authors did a thorough explanation on the methods employed, which included the development of a code book at priori, and the training of auditors. While disappointing to see yet another example of the lack of integration of SGIM in the preclinical curriculum, the authors highlighted some opportunities for curricular change, including: 1) over-representation of women in stigmatized diseases 2) lack of attention to anatomical of physiological differences in content topics, 3) the paucity of discussion of social, cultural or economic considerations. As a possible solution, the authors propose the creation of SGIM material in the form of slides, which could be integrated into the classes and collaborating with HMS-affiliated hospitals to deliberately integrate this content. I hope the authors follow up on this and report on the success of their efforts.

 Minor comments:

 The authors should revise the Figure Legend #2 describing what the colors within the bars mean.

Have any limitations of the research been acknowledged?

Yes

Is the work clearly and accurately presented and does it cite the current literature?

Yes

If applicable, is the statistical analysis and its interpretation appropriate?

Not applicable

Are all the source data underlying the results available to ensure full reproducibility?

Yes

Is the study design appropriate and is the work technically sound?

Yes

Are the conclusions drawn adequately supported by the results?

Yes

Are sufficient details of methods and analysis provided to allow replication by others?

Yes

Reviewer Expertise:

Neuroscience, aging, Women's Health

I confirm that I have read this submission and believe that I have an appropriate level of expertise to confirm that it is of an acceptable scientific standard.

# Response

**Published:** 2025-12-06

**Authors:** Deborah Bartz

**Affiliations:** Obstetrics and Gynecology, Brigham and Women's Hospital, Boston, Massachusetts, USA

We have recreated the figure to respond to reviewer 1 and it now has a key to define the colors noted in the columes
